# Additional risk factors for infection by multidrug-resistant pathogens in healthcare-associated infection: a large cohort study

**DOI:** 10.1186/1471-2334-12-375

**Published:** 2012-12-26

**Authors:** Teresa Cardoso, Orquídea Ribeiro, Irene César Aragão, Altamiro Costa-Pereira, António Eugénio Sarmento

**Affiliations:** 1 Unidade de Cuidados Intensivos Polivalente, Hospital Geral de Santo António, University of Porto, Largo Prof. Abel Salazar, 4099-001, Porto, Portugal; 2 Department of Health Information and Decision Sciences, Center for Research in Health Technologies and Information Systems (CINTESIS), Faculty of Medicine, University of Porto, Alameda Prof. Hernâni Monteiro, 4200-319, Porto, Portugal; 3 Department of Infectious Diseases, Hospital de São João, University of Porto, Alameda Prof. Hernâni Monteiro, 4200-319, Porto, Portugal

**Keywords:** Healthcare-associated infections, Multidrug resistant pathogens infection, Multidrug resistant gram negatives infection, ESKAPE microorganisms’ infection, Independent risk factors, Inadequate antibiotic therapy

## Abstract

**Background:**

There is a lack of consensus regarding the definition of risk factors for healthcare-associated infection (HCAI). The purpose of this study was to identify additional risk factors for HCAI, which are not included in the current definition of HCAI, associated with infection by multidrug-resistant (MDR) pathogens, in all hospitalized infected patients from the community.

**Methods:**

This 1-year prospective cohort study included all patients with infection admitted to a large, tertiary care, university hospital. Risk factors not included in the HCAI definition, and independently associated with MDR pathogen infection, namely MDR Gram-negative (MDR-GN) and ESKAPE microorganisms (vancomycin-resistant *Enterococcus faecium*, methicillin-resistant *Staphylococcus aureus*, extended-spectrum beta-lactamase-producing *Escherichia coli* and *Klebsiella* species, carbapenem-hydrolyzing *Klebsiella pneumonia* and MDR *Acinetobacter baumannii, Pseudomonas aeruginosa, Enterobacter* species), were identified by logistic regression among patients admitted from the community (either with community-acquired or HCAI).

**Results:**

There were 1035 patients with infection, 718 from the community. Of these, 439 (61%) had microbiologic documentation; 123 were MDR (28%). Among MDR: 104 (85%) had MDR-GN and 41 (33%) had an ESKAPE infection. Independent risk factors associated with MDR and MDR-GN infection were: age (adjusted odds ratio (OR) = 1.7 and 1.5, *p* = 0.001 and *p* = 0.009, respectively), and hospitalization in the previous year (between 4 and 12 months previously) (adjusted OR = 2.0 and 1,7, *p* = 0.008 and *p* = 0.048, respectively). Infection by pathogens from the ESKAPE group was independently associated with previous antibiotic therapy (adjusted OR = 7.2, *p* < 0.001) and a Karnofsky index <70 (adjusted OR = 3.7, *p* = 0.003). Patients with infection by MDR, MDR-GN and pathogens from the ESKAPE group had significantly higher rates of inadequate antibiotic therapy than those without (46% *vs* 7%*,* 44% *vs* 10%*,* 61% vs 15%, respectively, *p* < 0.001).

**Conclusions:**

This study suggests that the inclusion of additional risk factors in the current definition of HCAI for MDR pathogen infection, namely age >60 years, Karnofsky index <70, hospitalization in the previous year, and previous antibiotic therapy, may be clinically beneficial for early diagnosis, which may decrease the rate of inadequate antibiotic therapy among these patients.

## Background

Traditionally, infections have been classified as community or hospital-acquired, according to their place of acquisition, and this classification is still used to guide diagnosis and treatment of infections [1,2]. However, an increasing number of patients reside in nursing homes, the use of aggressive medical therapies (intravenous therapy, wound dressing) at home is more common, an increasing number of invasive therapies (hemodialysis, chemotherapy, radiotherapy) are performed in outpatient clinics, and there is a greater population of older patients, with more chronic diseases and frequent utilization of medical resources. This has led to the creation of a new group among the traditional classification of infections, termed “healthcare-associated infections” (HCAI).

In 2002, Deborah Friedman and colleagues [3] proposed a definition of HCAI including the above subgroups of patients, but despite being widely used in clinical studies [4-9], there is a lack of consensus regarding risk factors, and more recent studies have included additional risk factors such as an immunocompromised state, hospitalization in the previous year and prior antibiotic therapy [5,10]. Unfortunately, most of the studies performed using the HCAI classification have been restricted to respiratory infections [4,5,8,10], bloodstream infections [3,6,11] or a single pathogenic agent [7,9,12], creating the need to widen the study of risk factors for infection by multidrug-resistant (MDR) pathogens to all infected patients hospitalized from the community.

The objective of this study was to identify additional risk factors associated with infection by MDR pathogens in all infected patients admitted from the community into the hospital setting (patients with community-acquired and HCAI, including all foci of infection and associated pathogens), and not included in the adopted definition of HCAI [3].

## Methods

### Study design and patient population

A prospective cohort study was conducted in a 600-bed tertiary care university hospital, over a 1-year period (1 June 2008 to 31 May 2009). All consecutive adult infected patients admitted to the medical, surgical, nephrology or hematology wards of the hospital, or to the intensive care unit (ICU) were included, based on a diagnosis of infection according to the Center for Disease Control (CDC) criteria [1]. The primary outcome of interest was infection by a MDR pathogen. All patients completed the follow-up until hospital discharge. This study was approved by the Institutional Review Board of Hospital de Santo António, Oporto Hospital Centre, Portugal, and informed consent was waived owing to the observational nature of the study.

### Definitions

Community-acquired infection (CAI) was defined as an infection detected within 48 h of hospital admission in patients who did not fit the criteria for a HCAI.

HCAI was defined using the same criteria of Friedman et al. [3]-an infection present at the time of hospital admission or within 48 h of admission in patients that fulfilled any of the following criteria:

received intravenous therapy at home; received wound care or specialized nursing care through a healthcare agency, family or friends; or self-administered intravenous medical therapy in the 30-day period before the onset of the infection;

attended a hospital or hemodialysis clinic, or received intravenous chemotherapy in the previous 30 days;

hospitalized in an acute care hospital for 2 or more days in the previous 90 days;

resided in a nursing home or long-term care facility.

Hospital-acquired infection (HAI) was defined as a localized or systemic condition that resulted from an adverse reaction to the presence of an infectious agent(s) or its toxin(s), and that occurred 48 h or more after hospital admission and was not incubating at the time of admission [1]. Infections in patients recently discharged from hospital within the previous 2-week period were also included in this group.

The CDC definitions were used to define infections at different anatomic sites [1]. The definition of MDR organisms used was adopted from the CDC recommendations for the management of MDR organisms in healthcare settings that defines MDR organisms as bacteria that are resistant to one or more classes of antimicrobial agents recommended as first line therapy [13]. Thus, enteric Gram-negative rods were considered MDR (MDR-GN) if they were resistant to amoxicillin-clavulanate, piperacillin-tazobactam, carbapenems, aztreonam, fluoroquinolones, or third-generation cephalosporins or aminoglycosides. *Acinetobacter spp.* and *Pseudomonas spp.* were considered MDR if they were resistant to piperacillin-tazobactam, imipenem/meropenem, aztreonam, ciprofloxacin, cefepime, ceftazidime, aminoglycosides or colistin.

We grouped vancomycin-resistant *Enterococcus faecium*, methicillin-resistant *Staphylococcus aureus* (MRSA), extended-spectrum beta-lactamase (ESBL)-producing *Escherichia coli* and *Klebsiella* species, carbapenem-hydrolyzing *Klebsiella pneumonia* and MDR *Acinetobacter baumannii, Pseudomonas aeruginosa* and *Enterobacter* species into a group denominated ESKAPE, according to a previous publication of the Infectious Diseases Society of America [14].

The presence of ESBL production among *E. coli* and *Klebsiella spp.* strains was screened by the automatic analyzer Vitek2 (bioMérieux, France). It was confirmed by a disk diffusion test that detects synergism between the cephalosporins/monobactam and clavulanate. If the interpretation of the results was doubtful, we also performed the Etest® (AB Biodisk, Solna, Sweden); a combination of cefotaxime and cefotaxime/clavulanate and ceftazidime and ceftazidime/clavulanate indicated ESBL production whenever the ESBL ratio with antibiotic:antibiotic+inhibitor was ≥8.

The presence of carbapenemase production in *Enterobacteriaceae* was suspected whenever minimum inhibitory concentrations for ertapenem, imipenem and meropenem exceeded 0.5, 1 and 1 μg/mL, respectively. In such cases, a modified Hodge test was performed, and the ultimate confirmatory test was carbapenemase detection by molecular methods.

The risk factors studied for association with infection by MDR pathogens (including MDR-GN and the ESKAPE group) included age, sex, previous antibiotic therapy (in the last month prior to the current infection), previous hospitalization (in the last 12 months prior to the current infection, but not in the last 3 months), patient comorbidities and general medical condition. The comorbidities studied included immunosuppression (administration of radiation therapy in the 12 months prior to hospital admission, administration of 0.2 mg/kg/day of prednisolone for at least 3 months prior to hospital admission, administration of 1 mg/kg/day of prednisolone for 1 week in the 3 months prior to hospital admission or infection with human immunodeficiency virus), chronic liver disease [15], chronic heart failure [15], chronic respiratory disease [15], hematological disease [16], cancer (metastatic disease not under chemotherapy in the previous 12 months), diabetes mellitus requiring insulin therapy or oral hypoglycemic agents before the infection and/or atherosclerosis (defined as a previous history of a transient ischemic attack, stroke, angina, myocardial infarction or peripheral arterial disease). The patient’s general medical condition was assessed by the Karnofsky index [17]. A score of lower than 70 implies that the patient is unable to perform normal activities or do active work.

Adequacy of initial antibiotic therapy, hospital length of stay, Simplified Acute Physiology Score (SAPS II) and hospital mortality were also compared between groups of patients with and without infection by MDR pathogens (MDR-GN and ESKAPE). The initial empirical antibiotic treatment was considered “adequate” if the response to the initial antibiotic prescribed within 24 h of diagnosis matched *in vitro* susceptibility of the pathogen deemed to be the likely cause of the infection, and when the dosage and route of administration were appropriate for the patient’s current medical status (focus and severity of infection); only patients with positive microbiology were considered in this analysis. SAPS II [16] was recorded on the first day of antibiotic therapy.

### Statistical analysis

Continuous variables are described as mean and standard deviation (SD), or as median and inter-quartile range if they showed a skewed distribution. Categorical variables are described with absolute frequencies and percentages. Comparison of demographic and clinical characteristics of infected patients from the community (either with CAI or HCAI) and patients with HAI was made using the Student *t*-test (for continuous variables with normal distribution), the Mann–Whitney *U* test (for continuous variables with skewed distribution) and the Pearson *χ*^2^ test (for categorical variables).

Comparison of inadequate antibiotic therapy, hospital length of stay, SAPS II and hospital mortality between the group of patients infected with MDR pathogens and those not infected, along with comparison of the group infected by MDR-GN with those not infected, and the group infected by ESKAPE pathogens and those not infected, was made with the same tests according to the type of variables compared (continuous with normal distribution, continuous with skewed distribution or categorical).

All variables potentially associated with MDR pathogen infection (including MDR-GN and ESKAPE pathogens) were studied among all infected patients admitted from the community, those with CAI and HCAI, and included: age, sex, previous antibiotic therapy, hospitalization in the previous year, immunosuppression, chronic hepatic disease, chronic heart failure, chronic respiratory disease, chronic hematologic disease, cancer, diabetes, atherosclerosis and decreased functional capacity (Karnofsky index <70). Those with a clear association in the univariate analysis (*p* < 0.1) were included in the multivariable analysis. The results of the multivariable models are expressed as odds ratio (OR) with 95% confidence interval and *p*-values. The calibration was tested using the Hosmer-Lemeshow goodness-of-fit test. The significance level was defined as *p* < 0.05. Data were analyzed using SPSS version 18 for Windows (SPSS Inc., Chicago, IL, USA).

## Results

During the study period a total of 3733 patients were assessed and 1035 (28%) met the inclusion criteria of having infection according to the CDC definitions of infection. The mean ± SD age of the patients included was 65 ± 20 years, and the mean SAPS II was 29 ± 13; 49% of patients were male, and the overall hospital mortality rate was 13% (Table 1). Of these, 718 were admitted to hospital from the community with infection: 493 (48%) with a CAI and 225 (22%) with a HCAI. These populations were significantly different from patients with HAI, with a lower proportion of immunosuppressed patients (19% *vs* 27%, *p* = 0.007), a lower proportion of hospitalizations in the previous year (33% *vs* 55%, *p* < 0.001) and less likelihood of antibiotic therapy in the last month (19% *vs* 73%, *p* < 0.001). Hospital mortality was significantly lower in patients from the community (CAI or HCAI) than in those with HAI (11% *vs* 19%, *p* < 0.001) (Table 1). Of the 718 patients with CAI or HCAI, 439 (61%) had microbiologic documentation; of these, 123 were MDR (28%), 104 (85%) with MDR-GN and 41 (33%) with ESKAPE organisms.

**Table 1:** Demographic and clinical characteristics of patients, according to place of acquisition of infection

**Patients’ characteristics**	**TOTAL (n = 1035)**	**CAI and HCAI (n = 718)**	**HAI (n = 317)**	***p-value***
Male sex, n (%)	506 (49)	344 (48)	162 (51)	0.343*
Age, mean (SD)	65 (20)	65 (20)	64 (19)	0.405^&^
SAPSII, mean (SD)	29 (13)	29 (12)	30 (13)	0.962^&^
Severity of infection, n (%)				0.922*
Localized infection without SIRS	281 (27)	195 (27)	86 (27)	
Sepsis	364 (35)	251 (35)	113 (36)	
Severe sepsis	296 (29)	209 (29)	87 (27)	
Septic shock	94 (9)	63 (9)	31 (10)	
Previous comorbidities, n (%)	671 (65)	460 (64)	211 (67)	0.438*
Chronic hepatic disease, n (%)	22 (2)	18 (3)	4 (1)	0.822^$^
Chronic renal disease, n (%)	149 (14)	94 (13)	55 (17)	0.072*
Chronic heart failure, n (%)	74 (7)	46 (6)	28 (9)	0.163*
Chronic respiratory disease, n (%)	66 (6)	52 (7)	14 (4)	0.086*
Hematologic disease, n (%)	60 (6)	35 (5)	25 (8)	0.056*
Cancer, n (%)	45 (4)	26 (4)	19 (6)	0.084*
Diabetes, n (%)	204 (20)	144 (20)	60 (19)	0.674*
Atherosclerotic disease, n (%)	242 (23)	168 (23)	74 (23)	0.985*
Immunosuppression, n (%)	221 (21)	137 (19)	84 (27)	0.007*
Karnofsky index<70, n (%)	319 (31)	227 (32)	92 (29)	0.405*
Hospitalization in the previous year, n (%)	413 (40)	239 (33)	174 (55)	<0.001*
Previous antibiotic therapy, n (%)	367 (36)	137 (19)	230 (73)	<0.001*
Hospital mortality, n (%)	138 (13)	79 (11)	59 (19)	0.001*

CAI – community-acquired infection, HCAI – healthcare-associated infection, HAI – hospital-acquired infection, IQR – Inter-quartile range, SD – Standard deviation, SOFA – Sepsis-related Organ Failure Assessment, SIRS – Systemic inflammatory response syndrome.

*Pearson Qui-square Test; ^$^ Fisher exact test; & Independent samples *t-*test.

From the univariate analysis, age, previous antibiotic therapy, hospitalization in the previous year (from 4 months to 1 year), chronic heart failure, chronic respiratory disease, atherosclerosis and a Karnofsky index <70 were selected for multivariate analysis of independent risk factors associated with infection by MDR pathogens, including infection by MDR-GN (Table 2). For the ESKAPE group, previous antibiotic therapy, hospitalization in the previous year, and a Karnofsky index <70 were the variables selected for the multivariate model (Table 2).

**Table 2:** Risk factors for MDR, MDR gram-negatives and ESKAPE group pathogens infection

**Variables**	**Total, n (%) n=718**	**Microbiological documentation, n (%) n=439**	**MDR, n (%) n=123**	**Crude OR**	**p-value**	**MDR-GN, n (%) n=104**	**Crude OR**	**p-value**	**ESKAPE pathogens, n(%) n=41**	**Crude OR**	**p-value**
Age				1.6*	<0.001		1.5*	0.009		1.3*	0.170
≤60 years	271 (38)	166 (38)	33 (27)			30 (28)			13 (32)		
61-80 years	250 (35)	157 (36)	44 (36)			37 (36)			13 (32)		
>80 years	197 (27)	116 (26)	46 (37)			37 (36)			15 (36)		
Male sex	344 (48)	203 (46)	56 (46)	1.1	0.656	45 (43)	0.9	0.542	21 (51)	1.2	0.503
Previous antibiotic therapy	137 (19)	94 (21)	38 (31)	1.9	0.009	30 (29)	1.7	0.033	20 (49)	4.2	<0.001
Hospitalization in the previous year (4 months - 1 year)	146 (20)	102 (23)	37 (30)	2.1	0.006	29 (28)	1.8	0.031	14 (34)	3.3	0.003
Immunosupression (not meeting criteria of HCAI)	113 (16)	81 (19)	25 (20)	1.1	0.685	24 (23)	1.5	0.116	10 (24)	1.5	0.306
Chronic hepatic disease	18 (3)	14(3)	4 (3)	0.9	0.818	3 (3)	0.9	0.829	2 (5)	1.7	0.522
Chronic heart failure	46 (6)	27 (6)	13 (11)	2.5	0.026	11 (11)	2.3	0.038	2 (5)	0.8	0.723
Chronic respiratory disease	52 (7)	30 (7)	13 (11)	2.2	0.056	11 (11)	1.9	0.093	4 (10)	1.6	0.439
Chronic haematologic disease	35 (5)	29 (7)	8 (7)	1.0	0.945	5 (5)	0.7	0.444	5 (12)	2.2	0.139
Cancer (not meeting criteria of HCAI)	10 (1)	6 (1)	2 (2)	1.5	0.666	2 (2)	1.6	0.578	0 (0)		
Diabetes	144 (20)	96 (22)	32 (26)	1.3	0.291	27 (26)	1.4	0.239	11 (27)	1.4	0.421
Atherosclerosis	168 (23)	105 (24)	40 (33)	1.8	0.017	32 (31)	1.6	0.069	13 (32)	1.5	0.222
Karnofsky index<70	227 (32)	149 (34)	57 (46)	1.8	0.006	44 (42)	1.6	0.046	22 (54)	2.5	0.006

Association of demographic and clinical variables with infection by MDR, MDR gram negatives and ESKAPE pathogens among patients admitted from the community (with community-acquired or healthcare-associated infections), using logistic regression.

*increase in the odds per category of age; MDR – multi-drug resistant; MDR-GN – multi-drug resistant gram negatives; OR – odds ratio; ESKAPE - Vancomycin-resistant *Enterococcus faecium*, MRSA, ESBL producing *E. coli* and *Klebsiella* species, *Klebsiella pneumonia* Carbapenamase-hydrolyzing and MDR *Acinectobacter baumannii, Pseudomonas aeruginosa* and *Enterobacter* species.

Regarding infection by MDR pathogens, the final model retained age (adjusted OR per category = 1.7, *p* = 0.001) and hospitalization in the previous year (from 4 months to 1 year) (adjusted OR = 2.0, *p* = 0.008) (Table 3) as independent risk factors. The final model for MDR-GN infection also retained age (adjusted OR per category = 1.5, *p* = 0.009) and hospitalization in the previous year (from 4 months to 1 year) (adjusted OR = 1.7, *p* = 0.048) (Table 3). For ESKAPE pathogen infection, the final model retained previous antibiotic therapy (adjusted OR = 7.2, *p* < 0.001) and Karnofsky index <70 (adjusted OR = 3.7, *p* = 0.003) (Table 3).

**Table 3:** Independent variables associated with infection by MDR, MDR gram-negatives and pathogens from the ESKAPE group

**Variables**	**MDR, n (%) n = 123**	**Adjusted OR**	**CI**_**95%**_	**p-value**	**MDR Gram negatives, n (%) n = 104**	**Adjusted OR**	**CI**_**95%**_	**p-value**	**ESKAPE pathogens, n(%) n= 41**	**Adjusted OR**	**CI**_**95%**_	**p-value**
Age		1.7	1.3-2.3	0.001		1.5	1.1-2.1	0.009				
≤60 years	33 (27)				30 (28)				13 (32)			
61-80 years	44 (36)				37 (36)				13 (32)			
>80 years	46 (37)				37 (36)				15 (36)			
Previous antibiotic therapy	38 (31)				30 (29)				20 (49)	7.2	3.1-17.0	<0.001
Hospitalization in the previous year (4 months - 1 year)	37 (30)	2.0	1.2-3.4	0.008	29 (28)	1.7	1.0-3.0	0.048	14 (34)			
Karnofsky index<70	57 (46)				44 (42)				22 (54)	3.7	1.6-8.6	0.003

Selection of variables significantly and independently associated with infection by a MDR pathogen, MDR gram negative or a pathogen from the ESKAPE group, among the group of patients admitted from the community (with community or healthcare-associated infection), using multiple logistic regression.

*increase in the odds per category of age; MDR – multi-drug resistant; OR – odds ratio; CI – confidence interval; ESKAPE - Vancomycin-resistant *Enterococcus faecium*, MRSA, ESBL producing *E. coli* and *Klebsiella* species, *Klebsiella pneumonia* Carbapenamase-hydrolyzing and MDR *Acinectobacter baumannii, Pseudomonas aeruginosa* and *Enterobacter* species.

Patients with infection by a pathogen belonging to one of the three MDR groups had a significantly higher rate of inadequate antibiotic therapy: 16% in the study population, 46% in the group of patients with infection by MDR pathogen, 44% in the group with infection by MDR-GN, and 61% in the group with an ESKAPE group infection (*p* < 0.001). Despite that, median hospital length of stay was similar in all groups, as were SAPS II and hospital mortality rate (Table 4).

**Table 4:** Impact of infection by MDR pathogens, including MDR gram-negatives and pathogens from the ESKAPE group, in antibiotic therapy and hospital outcome

**Variables**	**MDR,**	**Non-MDR,**	**Non- MDR*vs*MDR,**	**MDR-GN**	**Non-MDR-GN,**	**Non-MDR-GN*vs*MDR-GN,**	**ESKAPE pathogens,**	**Non-ESKAPE pathogens,**	**Non-ESKAPE*vs*ESKAPE,**
	**n (%)**	**n (%)**	**p Value**	**n (%)**	**n (%)**	**p Value**	**n (%)**	**n (%)**	**p value**
Inadequate ATB therapy	57 (46)	19 (7)	<0.001*	46 (44)	30 (10)	<0.001*	25 (61)	51 (15)	<0.001*
Hospital LOS, median (IQR)	10 (7–17)	10 (7–16)	0.944^#^	10 (7–15)	10 (7–17)	0.487^#^	11 (8–15)	10 (7–17)	0.280^#^
SAPS II, mean (SD)	32 (13)	30 (13)	0.171^&^	31 (13)	30 (13)	0.428^&^	32 (17)	30 (12)	0.373^&^
Hospital mortality	19 (15)	31 (12)	0.280*	13 (13)	37 (13)	0.928*	9 (22)	41 (12)	0.062*

Comparison of inadequate antibiotic therapy, hospital length of stay, SAPS II and hospital mortality between the group of patients infected with MDR pathogens and those not infected, along with comparison of the group infected by MDR-GN with those not infected, and the group infected by ESKAPE pathogens and those not infected.

ATB – antibiotherapy, CAI – community-acquired infection, LOS – length of stay, IQR – inter-quartil range, MDR – multi-drug resistant, MDR GN – multi-drug resistant gram negatives, ESKAPE - Vancomycin-resistant *Enterococcus faecium*, MRSA, ESBL producing *E. coli* and *Klebsiella* species, *Klebsiella pneumonia* Carbapenamase-hydrolyzing and MDR *Acinectobacter baumannii, Pseudomonas aeruginosa* and *Enterobacter* species.

*Pearson Qui-square Test; # Mann–Whitney test, &Students *t-*test.

## Discussion

In this study, age >60 years, hospitalization in the previous year (in the last 4 to12 months), previous antibiotic therapy (last month) and Karnofsky index <70 were found to be significantly and independently associated with infection by MDR pathogens. These risk factors were not considered in the definition of risk factors of HCAI proposed by Friedman et al. Patients with infection by MDR microorganisms had a significantly higher rate of inadequate initial antibiotic therapy, but did not have a longer hospital length of stay or higher hospital mortality rate. This is probably one of the first studies containing a thorough description of independent and significant risk factors associated with infection by pathogens of the ESKAPE group as well as their impact regarding inadequate antibiotic therapy, hospital length of stay and hospital mortality.

In order to define the new group of infections across community and hospital settings, a definition of all risk factors for MDR pathogen infections is required. The definition proposed by Friedman et al. [3] is one of the most used in clinical studies regarding HCAI, but it was developed for bloodstream infections. Further studies followed, using similar definitions, but again only for a single focus of infection or single pathogens. Our study identified additional risk factors for MDR pathogen infection.

Micek et al. [10] performed a retrospective cohort study of hospitalized patients with pneumonia, and identified immunocompromised state and hospitalization in the previous 12 months as risk factors for HCAI. They found a higher proportion of MDR organisms in the HCAI group, with a significant association with inappropriate antibiotic therapy and hospital mortality rate. One limitation discussed by the authors was the fact that they did not evaluate severity-of-illness scores. In the current study severity scores were considered and they were similar in all groups, with no significant differences in hospital mortality rate between groups. Park et al. [5] also performed a retrospective cohort study on HCAI-pneumonia, using the Friedman et al. definition and prior antibiotic therapy in the 30 days before the pneumonia episode, and found a higher proportion of MDR pathogens when compared with community-acquired pneumonia, and a significant association with inappropriate initial antibiotic therapy. Brito and Niederman [18] performed a review of all articles published since the American Thoracic Society guidelines were published [2], and concluded that patients at risk of pneumonia by MDR pathogens were those who had severe illness, were hospitalized in the past 90 days, had antibiotic therapy in the past 6 months, had poor functional status as defined by activities of daily living score, and were immunosuppressed, similar to the risk factors described here.

Wright et al. [19] performed a retrospective cohort study to determine risk factors for MDR pathogens among patients in the Emergency Department with urinary tract infections, and described the following independent risk factors: age ≥65 years, diabetes, prior use of antibiotics, and urinary catheterization. Chen et al. [20] studied patients from the community with bacteremia but with prior hospitalization from periods of 3–90, 91–180 to up to 181–360 days, and concluded that the presence of antimicrobial-resistant bacteria in the past 360 days was an independent risk factor for antimicrobial-resistant bacteremia, reaffirming that hospitalization in the previous year represented a risk factor for MDR pathogen infection. Pop-Vicas et al. [21], in a 6-year study of patients with positive cultures for MDR organisms, noted an increasing prevalence of MDR-GN at admission to a tertiary-care hospital (positive cultures within the first 48 h after hospital admission). Factors independently associated with isolation of the resistant organisms were age ≥65 years, prior antibiotic therapy for ≥14 days and prior residence in a long-term-care facility.

The current study has the major advantage of being prospective, with clear and consensual definition of infection [1]. It included all major foci of infection that drove patients into hospital care and not only pneumonia, bacteremia or urinary infection as in other previous studies. It clearly identified the place of acquisition and the appropriateness of initial antibiotic therapy. Data regarding severity of illness and the impact of infection by MDR organisms in the appropriateness of initial antibiotic therapy, hospital length of stay and mortality rate were also investigated. Data collection was performed by a single trained doctor, and all protocols were completed with no missing data thus minimizing information bias. All patients completed follow-up until hospital discharge. Most importantly, risk factors described in this study have been previously mentioned in studies restricted to single focus of infection or pathogen, by other authors from different parts of the world, suggesting important external applicability of these conclusions.

The study also has certain limitations that should be acknowledged. The research was performed in a single institution and the number of patients from the community was relatively small. There is accumulating evidence that HCAI seems to be a group encompassing CAI and HAI, but specific definition of risk factors are lacking. The diversity of definitions used has led to diverse findings not comparable between studies, and therefore not consequential for specific treatment recommendations.

Implementation of standardized approaches for the treatment of HCAI is desirable, since failure to recognize this group of infected patients appears to be associated with administration of inappropriate antibiotic therapy in hospitalized patients with serious infection, and may be related to poor prognosis.

## Conclusions

The results of this study suggest that the inclusion of additional risk factors for MDR pathogen infection in HCAI definition, namely age >60 years, Karnofsky index <70, hospitalization in the previous year, and previous antibiotic therapy, may be clinically beneficial because of early diagnosis and a decrease in the rate of inadequate antibiotic therapy in these patients. Further research involving a large number of patients from different institutions and geographic areas is warranted to confirm these findings.

## Abbreviations

CAI: Community-acquired infection; HCAI: Healthcare-associated infection; HAI: Hospital-acquired infection; MDR: Multidrug-resistant; MDR-GN: Multidrug-resistant Gram-negative; ESKAPE: Vancomycin-resistant *Enterococcus faecium*, methicillin-resistant *Staphylococcus aureus* (MRSA), extended-spectrum beta-lactamase (ESBL)-producing *E. coli* and *Klebsiella* species, carbapenem-hydrolyzing *Klebsiella pneumonia* and MDR *Acinetobacter baumannii, Pseudomonas aeruginosa* and *Enterobacter* species; CDC: Center for Disease Control; ICU: Intensive Care Unit; MRSA: Methicillin-resistant *Staphylococcus aureus* ; ESBL: Extended-spectrum beta-lactamase; MIC: Minimum inhibitory concentration; SAPS: Simplified acute physiological score; IQR: Inter-quartile range; SD: Standard deviation; OR: Odds ratio; CI_95%_: 95% confidence interval.

## Competing interests

The authors declare that they have no competing interests.

## Authors’ contributions

TC, ACP and AS conceived the study. TC coordinated data collection. TC and OR performed the statistical analysis. All authors participated in the analysis and interpretation of the data, clinical revision of the manuscript and final approval of the version to be published.

## Pre-publication history

The pre-publication history for this paper can be accessed here:

http://www.biomedcentral.com/1471-2334/12/375/prepub
